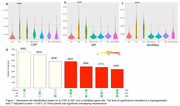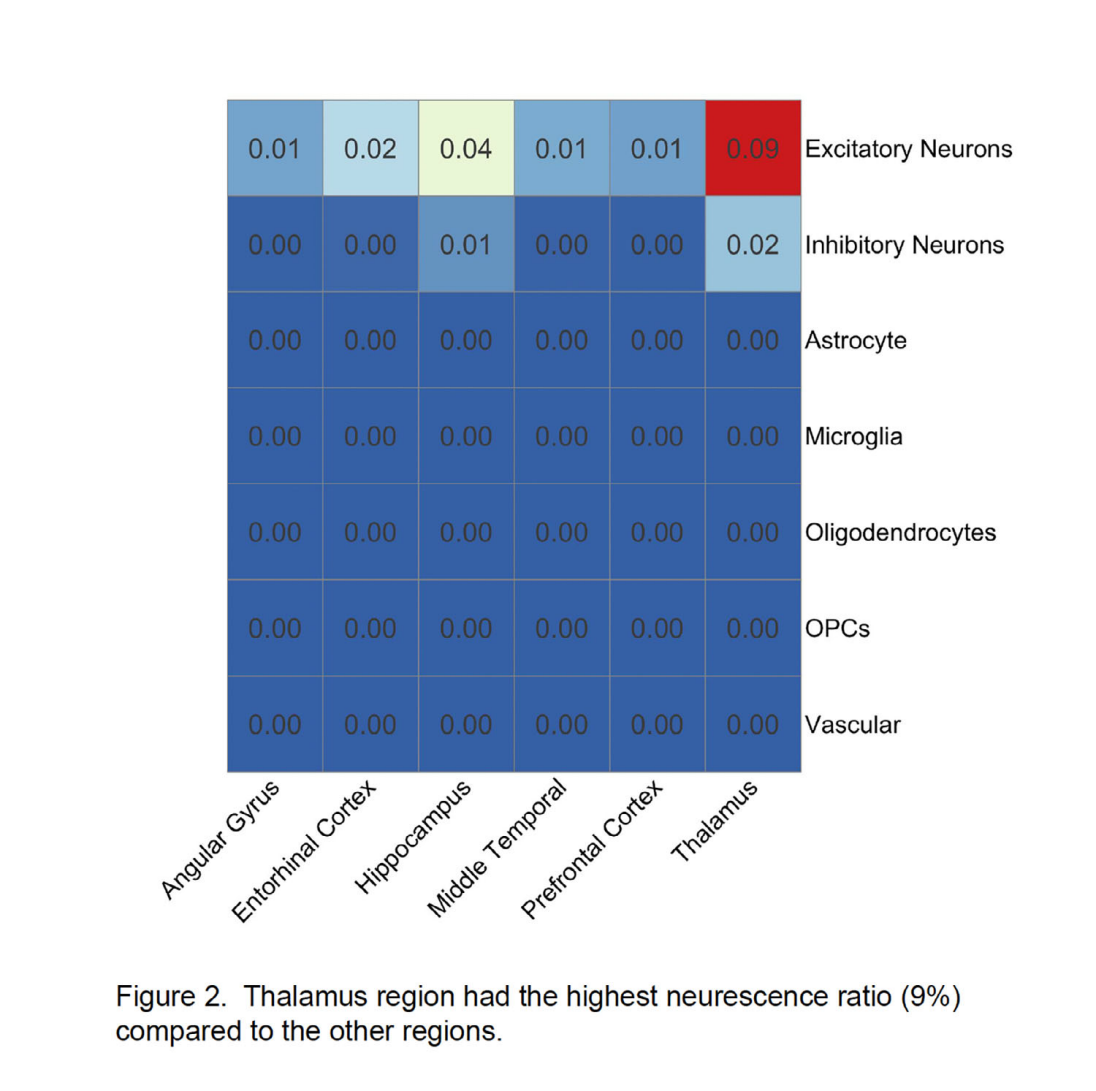# Thalamus has the highest density of neurescent among human brain regions

**DOI:** 10.1002/alz70855_104411

**Published:** 2025-12-24

**Authors:** Shiva Kazempour Dehkordi, Habil Zare, Miranda E. Orr

**Affiliations:** ^1^ University of Texas Health Science Center at San Antonio, San Antonio, TX, USA; ^2^ The University of Texas Health Science Center at San Antonio, San Antonio, TX, USA; ^3^ Washington University School of Medicine, St. Louis, MO, USA

## Abstract

**Background:**

Cellular senescence is a complex, multi‐step process and is recognized as a contributor to neurodegenerative diseases, including Alzheimer's disease (AD). This study is focused on neurescence (i.e., senescent neurons). Analyzing single nucleus (sn) RNA‐Seq data from postmortem human brain, we previously identified neurescent excitatory neurons in the dorsolateral prefrontal cortex, comprising 2% of the total cell population. The prevalence of senescent cells, including neurescence, in other brain regions affected by Alzheimer's disease remains unknown. This study aims to identify senescent cell types across six brain regions.

**Method:**

We evaluated snRNA‐Seq data from 48 postmortem brains (PMID: PMC11338834) to identify senescent cell types, utilizing the transcriptional profiles of around 1.3 million cells across six brain regions. We used an eigengene‐based approach, where an eigengene is a weighted average expression of genes that summarizes biological signatures with minimal loss of information (PMID: 28298217). We calculated eigengenes for key senescence pathways: a) the SenMayo gene list (PMID: PMC9381717) (125 genes), b) Canonical Senescence Pathway (CSP, 22 genes), which reflect cell cycle arrest, and c) Senescence Initiating Pathway panel (SIP, 48 genes), which are upregulated in early senescence and activate senescent cell anti‐apoptotic pathways (SCAPs). Cells were classified as 'senescent' if all three eigengenes exceeded the mean plus three standard deviations.

**Result:**

Excitatory neurons were the only cell type significantly associated with senescence across all regions (Figure 1a‐c). A substantial number of cells showed overlapping senescence signatures across the CSP, SIP, and SenMayo (Figure 1d), confirming the robustness of these pathways in identifying senescent cells and highlighting shared molecular features among these gene panels. Among the six brain regions analyzed, the thalamus exhibited the highest neurescence ratio (9%), significantly exceeding the ratios observed in the other regions (1%‐4%, Figure 2).

**Conclusion:**

Our findings highlight the thalamus as a critical brain region for investigating neurescence, with significantly higher neurescence ratios compared to other regions. This study is the first to report excitatory and inhibitory neurons as key cell types affected in the thalamus.